# Biological nitrification inhibitor-trait enhances nitrogen uptake by suppressing nitrifier activity and improves ammonium assimilation in two elite wheat varieties

**DOI:** 10.3389/fpls.2022.1034219

**Published:** 2022-11-11

**Authors:** Adrián Bozal-Leorri, Guntur V. Subbarao, Masahiro Kishii, Leyre Urmeneta, Víctor Kommerell, Hannes Karwat, Hans-Joachim Braun, Pedro Mª Aparicio-Tejo, Iván Ortiz-Monasterio, Carmen González-Murua, Mª Begoña González-Moro

**Affiliations:** ^1^ Department of Plant Biology and Ecology, Faculty of Science and Technology, University of the Basque Country (UPV/EHU), Bilbao, Spain; ^2^ Crop, Livestock and Environment Division, Japan International Research Center for Agricultural Sciences, Ibaraki, Japan; ^3^ Global Wheat Program, International Maize and Wheat Improvement Center, Texcoco, Mexico; ^4^ Institute for Multidisciplinary Research in Applied Biology (IMAB), Public University of Navarre, Pamplona, Spain

**Keywords:** ammonia-oxidizing archaea, ammonia-oxidizing bacteria, N fertilization, nitrogen use efficiency, synthetic nitrification inhibitor

## Abstract

Synthetic nitrification inhibitors (SNI) and biological nitrification inhibitors (BNI) are promising tools to limit nitrogen (N) pollution derived from agriculture. Modern wheat cultivars lack sufficient capacity to exude BNIs, but, fortunately, the chromosome region (Lr#n-SA) controlling BNI production in *Leymus racemosus*, a wild relative of wheat, was introduced into two elite wheat cultivars, ROELFS and MUNAL. Using BNI-isogenic-lines could become a cost-effective, farmer-friendly, and globally scalable technology that incentivizes more sustainable and environmentally friendly agronomic practices. We studied how BNI-trait improves N-uptake, and N-use, both with ammonium and nitrate fertilization, analysing representative indicators of soil nitrification inhibition, and plant metabolism. Synthesizing BNI molecules did not mean a metabolic cost since Control and BNI-isogenic-lines from ROELFS and MUNAL presented similar agronomic performance and plant development. In the soil, ROELFS-BNI and MUNAL-BNI plants decreased ammonia-oxidizing bacteria (AOB) abundance by 60% and 45% respectively, delaying ammonium oxidation without reducing the total abundance of bacteria or archaea. Interestingly, BNI-trait presented a synergistic effect with SNIs since made it also possible to decrease the AOA abundance. ROELFS-BNI and MUNAL-BNI plants showed a reduced leaf nitrate reductase (NR) activity as a consequence of lower soil ${\text{NO}}_{3}^{-}$ formation and a higher amino acid content compared to BNI-trait lacking lines, indicating that the transfer of Lr#-SA was able to induce a higher capacity to assimilate ammonium. Moreover, the impact of the BNI-trait in wheat cultivars was also noticeable for nitrate fertilization, with improved N absorption, and therefore, reducing soil nitrate content.

## 1 Introduction

Nitrogen (N) availability is the major nutrient condition limiting crop growth (LeBauer and Treseder, 2008). Hence, agriculture relies on the intensive use of N fertilizers to maximize crop yields, which is foretold to reach 300 Tg N year^-1^ by 2050 (Subbarao et al., 2022). Unfortunately, the entire N applied cannot be taken up by the crop or retained in the soil and, consequently, a great amount is lost as reactive N, causing a negative environmental impact. The main pathways for nitrogen losses are through nitrate ( ${\text{NO}}_{3}^{-}$ ) leaching, ammonia volatilization (NH_3_), and emissions of nitrogenous gases such as nitric oxide (NO) and nitrous oxide (N_2_O). The latter is the main greenhouse gas generated in upland agriculture derived from the use of N fertilizers (Syakila et al., 2011). It is estimated that agriculture is responsible for the emission of 1.7-4.8 Mg N_2_O-N year^-1^, representing around 19% of total N_2_O global source and 49% of anthropogenic N_2_O emissions (Fowler et al., 2009). N_2_O is generated by soil microorganisms through nitrification and denitrification processes (Li et al., 2016). Nitrification is the biological process that sequentially oxidizes ammonium ( ${\text{NH}}_{4}^{+}$ ) to hydroxylamine (NH_2_OH), nitrite ( ${\text{NO}}_{2}^{-}$ ), and eventually to nitrate ( ${\text{NO}}_{3}^{-}$ ) carried out by ammonia-oxidizing bacteria (AOB) and archaea (AOA), and nitrite-oxidizing bacteria (Arp and Stein, 2003; Könneke et al., 2005). During this process, N_2_O is released as a product during NH_2_OH oxidation to nitrite (Wrage et al., 2001). The formed ${\text{NO}}_{3}^{-}$ , if not rapidly taken up by plant roots, is susceptible to leaching or becoming the substrate for the denitrification process, releasing NO, N_2_O, and molecular nitrogen (N_2_) that return to the atmosphere (Hochstein and Tomlinson, 1988).

At present, available strategies for reducing N losses related to nitrification are through the application of synthetic nitrification inhibitors (SNIs) when applying ammonium-based fertilizers. Currently, the most commonly used SNIs worldwide are nitrapyrin (2-chloro-6-(tri-chloromethyl)-pyridine), dicyandiamide (DCD), and 3,4-dimethylpyrazole phosphate (DMPP) (Trenkel, 2010). The addition of SNIs delays ${\text{NH}}_{4}^{+}$ oxidation and reduces ${\text{NO}}_{3}^{-}$ leaching and N_2_O emissions (Ruser and Schulz, 2015). Nevertheless, the performance of these chemicals varies substantially depending on soil conditions, such as pH (Liu et al., 2015) or soil temperature, which can determine the half-life of SNIs and, therefore, their efficacy (Kelliher et al., 2008; Chen et al., 2010). Although SNIs are effective in reducing AOB growth, they have limited capacity to inhibit AOA growth (Beeckman et al., 2018). Limited adoption of SNI technology in production agriculture is due to a lack of cost-effectiveness and inconsistency in-field performance (Subbarao et al., 2017). Fortunately, the use of biological nitrification inhibitors (BNIs) shows as a promising option to alleviate N losses from nitrification. The phenomenon termed “biological nitrification inhibition” (BNI) refers to the natural ability of some plants to suppress soil nitrification by releasing allelochemical compounds from roots (Subbarao et al., 2015). *Brachiaria* grasses present the highest BNI-capacity; but sorghum (*Sorghum bicolor*) is at the forefront among crops in terms of BNI-production capacity (Subbarao et al., 2009; Subbarao et al., 2013). BNIs are exuded into the rhizosphere, a site with a greater abundance of AOB and AOA (Nardi et al., 2020); both of which are affected by BNIs (Nardi et al., 2013; Byrnes et al., 2017; Lu et al., 2019; Lan et al., 2022).

Wheat is the main crop used for human food and its production is expected to reach 3.8 Mg ha^-1^ by 2050 (Alexandratos and Bruinsma, 2012). Many wheat agrosystems are managed under intensive fertilization due to the high N requirement (Tilman et al., 2002). Unfortunately, modern wheat cultivars lack detectable BNI capacity in their root systems (Subbarao et al., 2007a) and require the application of SNIs or a more appropriate N management to reduce N pollution. Several works report a reduction in soil ${\text{NO}}_{3}^{-}$ formation and N_2_O emissions from wheat systems using SNIs or adequate N management, without affecting the yield (Matson et al., 1998; Weiske et al., 2001; Migliorati et al., 2014; Huérfano et al., 2015; Dawar et al., 2021). On the other hand, Subbarao et al. (2007b) reported that *Leymus racemosus*, a wild relative of wheat, has high-BNI capacity, and the genes responsible for this capacity were located on chromosomes *Lr#n*. With the idea of endowing wheat cultivars with BNI capacity, Subbarao et al. (2021) introduced the BNI-controlling chromosome region from *L. racemosus* into several elite high-yielding wheat cultivars. Authors accomplished near doubling the BNI-capacity in root systems of elite wheat cultivars “ROELFS-BNI” and “MUNAL-BNI” (Subbarao et al., 2021). Further, field studies using isogenic-lines of MUNAL (i.e. MUNAL-BNI vs MUNAL-Control) under slightly acidic soils (pH 6.0) indicate significant improvements in grain yields in BNI-trait harbouring wheat-line under wide-ranging N inputs. In addition, nitrification and N_2_O emissions from rhizosphere soils where MUNAL-BNI grew were significantly lower (about 30%) than in soils with MUNAL-Control (Subbarao et al., 2021). The development of these new BNI-producing wheat lines marks a major milestone along the path toward greater agrosystems sustainability since it would allow farmers to have highly productive wheat crops while reducing N fertilizer inputs and, consequently, reducing N leakage and environmental pollution.

This new technology could be used by many farmers all over the world. Nevertheless, before widespread use in wheat agrosystems, it is essential to validate the efficiency of the BNI-trait considering different edapho-climatic conditions, such as the type of soil, and different fertilizer types. Therefore, in this work, we considered relevant the inclusion of a field experiment performed at the International Maize and Wheat Improvement Center (CIMMYT), in Mexico, with two wheat lines outfitted with BNI-trait, MUNAL and ROELFS. CIMMYT holds its main wheat research station in Mexico at CENEB, in part because this location is agro-ecologically representative of Mega-environment one (ME1). The region ME1 comprises a group of locations where 40% of wheat is produced in developing countries (Rajaram et al., 1993). Therefore, the lessons learned from the wheat agrosystem in this location can be applicable to other regions of this mega-environment. In Northern Spain, in the Basque Country, wheat is cultivated in the province of Araba (Álava) under humid Mediterranean climatic conditions, where soils show alkaline pH values. Performing microcosm experiments in this location would fill the knowledge gap about the performance of BNI-trait wheat under non-acidic physicochemical soil conditions. BNI-wheat on acidic soil has been tested by Subbarao et al. (2021). Our research aimed to evaluate in field and microcosm if the BNI-trait expression is maintained in alkaline soils, and to which extent the use of different nitrogen sources, such as ammonium or nitrate, influences its expression. Since wheat is a relatively ammonium-tolerant crop (González-Moro et al., 2021), our experimental approach has also considered the application of DMPP, a widely used SNI (Trenkel, 2010), to test the hypothesis that ROELFS-BNI and MUNAL-BNI plants could be more favoured in environments with greater ammonium availability to understand the complementarity and/or the synergistic impact from BNI and SNI functions in limiting nitrogen leakage.

## 2 Materials and methods

### 2.1 Field experiment design and agronomic analysis

A field experiment was established at the Experimental Station Norman Borlaug CENEB (CIMMYT) at the Yaqui Valley, near Ciudad Obregón (Sonora, Mexico) in one growing season 2019/2020. Soil characteristics are described in **Supplementary Table 1**. The experiment was set up as a split-plot design with four replications, the main plot was fertilized with 250 kg N ha^-1^ single application as ammonium sulphate ((NH_4_)_2_SO_4_), and the subplots were the four genotypes from ROELFS and MUNAL isogenic-lines (ROELFS-Control, ROELFS-BNI, MUNAL-Control, and MUNAL-BNI). The experiment was also fertilized with 69 kg P_2_O_5_ ha^-1^ using triple super phosphate, applied pre-planting as a broadcast, and then incorporated. The experiment was set up within the optimum planting date, with a density of 250 seed m^-2^. The experiment received five irrigations through the crop cycle when available soil water reached 50% and all weeds, diseases, and insects were controlled. The experimental unit was four beds 75-cm apart and 5-m long. The harvest area was the 2 central beds and the central 3 meters and it was done using a Wintersteiger experimental plot combine. The total grain protein content was taken as 5.7 times the total N content (Teller, 1932), which was analysed by applying the Kjeldahl procedure.

### 2.2 Microcosm experimental design and plant material

This experiment was carried out in microcosms in a controlled conditions greenhouse at UPV/EHU (Bilbao, Spain) with a day/night cycle regimen of 14/10 h, average temperature of 25/18 °C, and relative humidity of 50/60%. Four elite wheat (*Triticum aestivum*) genetic stocks comprising two isogenic-lines for BNI-trait (ROELFS-Control, ROELFS-BNI, MUNAL-Control, MUNAL-BNI) were tested in this study (Subbarao et al., 2021). To germinate, 240 seeds per BNI-isogenic-line were placed in square Gosselin plates at 5°C in darkness for 7 days. Then, seeds were transferred to trays with perlite:vermiculite (1:3; v:v) mixture at 20°C for 4 days.

Soil was collected in June 2019, from a 0–30 cm layer of Hypercalcic Kastanozen soil (IUSS, 2015) with a pH value of 8.0 in a wheat field (**Supplementary Table 1**) in Arkaute (Araba, the Basque Country, Spain). Soil was passed through a 5-mm sieve after roots and stones were removed. Soil was mixed with sand in proportion of soil:sand (3:1, v:v) to increase soil porosity and to avoid compaction that would avert normal root development. Afterward, soil was air-dried, homogenised, and kept at 4° C until the start of the experiment. Sixty-four pots of 1.35 L (12.5-cm diameter x 17-cm height) were filled with the soil. In order to reactivate N-cycle soil microorganisms, an extra carbon source in form of glucose (1.1 mg glucose) and 86 mg ammonium sulphate ((NH_4_)_2_SO_4_) (Menéndez et al., 2012) were added to each pot, and the soil was rehydrated with deionised water up to 45% water filled pore space (WFPS). WFPS was calculated following the equation described in Linn and Doran (1984) (Eq. 1):


(1) $$\text{WFPS}=(C\times D_{\text{b}})\times {\left(1-(D_{\text{b}}/D_{\text{p}})\right)}^{-1}$$

where *C* (g) is the soil gravimetric water content, *D*_b_ (Mg m^-3^) is the bulk density; *D*_p_ (Mg m^-3^) is the particle density. *D*_b_ was determined in the laboratory, resulting in a value of 1.31 Mg m^-3^, while *D*_p_ was assumed at 2.65 Mg m^-3^. After 14 days of soil activation, pots were divided into 4 groups (one per Control and BNI-isogenic-lines of ROELFS and MUNAL respectively). Later, 4 seedlings of their corresponding Control and BNI-isogenic-line were placed in each pot, and the soil was watered for 15 days to maintain the WFPS up to 45% and to ensure nitrifying conditions.

On the 15^th^ day, 4 replicates per Control and BNI-isogenic-lines were harvested as T0 and the remaining pots were fertilized. Within each isogenic-line, 3 N-fertilization treatments (4 replicates per treatment) were established, as follows: 1) fertilization with ammonium sulphate (AS); 2) fertilization with ammonium sulphate + DMPP (AS+D); and 3) fertilization with potassium nitrate (KN). Nitrogen was applied in an equivalent dose to 195 kg N ha^−1^, which was achieved by adding 1726 mg ${\text{KNO}}_{3}^{-}$ or 1128 mg (NH_4_)_2_SO_4_, alone or mixed with DMPP (EuroChem Agro Iberia S.L.). DMPP content represented 0.8% of applied N. To achieve a homogeneous distribution of the nitrogen in the soil, the fertilizer was dissolved in deionised water and added to the corresponding pots by pipetting. All the treatments were watered every two days to maintain the 45% WFPS up to 30 days after fertilization, when the plants were between Z51 and Z53 stages (Zadoks et al., 1974). Following this time, wheat plants were harvested for physiological determinations or immediately frozen in liquid N for biochemical and metabolic measurements. Soil was sampled in parallel and dried for 48 h at 70° C.

#### 2.2.1 Soil analysis

The abundance of nitrifying and denitrifying genes in soil was quantified through quantitative polymerase chain reaction (qPCR). Dry soil (0.25 g) was used to extract the DNA, using the PowerSoil DNA Isolation Kit (Quiagen) with the modifications described in Harter et al. (2014). For quantification of total bacterial and archaeal abundance *16 rRNA* gene was used and genes involved in nitrification (bacterial and archaeal *amoA*) and denitrification (*nirK*, *nirS*, *nosZI*, and *nosZII*) were amplified as described by Bozal-Leorri et al. (2022).

Soil mineral N was determined as ${\text{NH}}_{4}^{+}$ and ${\text{NO}}_{3}^{-}$ contents. Aliquots of fresh soil (100 mg) were mixed with 1 M KCl (200 mL) and shaken at 165 rpm for one hour. The soil solution was filtered, firstly through Whatman n°1 filter paper (GE Healthcare) and secondly through Sep-Pak Classic C18 Cartridges 125 Å-pore size (Waters), to remove particles and organic matter respectively. The Berthelot method was followed to quantify the ${\text{NH}}_{4}^{+}$ content (Patton and Crouch, 1977). The ${\text{NO}}_{3}^{-}$ content was determined according to Cawse (1967).

#### 2.2.2 Plant determinations and enzymatic activity

Biomass production was given as dry weight (DW) per plant. To do so, one plant per pot was dried at 80°C in a circulation oven for 72 h until a constant DW was reached.

Leaf ${\text{NH}}_{4}^{+}\text{, }{\text{NO}}_{3}^{-}$ , and total amino acid content were quantified from 50 mg of frozen leaf powder. Plant material was homogenized with 1 mL Milli-Q^®^ water in a ball miller (Retsch MM 500) for 3 min at a frequency of 27 s^-1^. Homogenates were incubated at 80 °C for 5 min and, afterward, centrifuged at 16,000 *g* for 20 min. Later, supernatants were recovered and stored at -20°C until metabolite quantification. ${\text{NH}}_{4}^{+},\;{\text{NO}}_{3}^{-}$ and total amino acid content were determined in the supernatants as described in Cataldo et al. (1975); Patton and Crouch (1977), and Yemm et al. (1955), respectively.

Nitrate reductase (NR) activity was determined in leaves by the modified *in vivo* method (Jaworski, 1971; Ligero et al., 1987; Karwat et al., 2019). The NR activity was determined in the flag leaf, which was harvested between Z51 and Z53 stages (Zadoks et al., 1974). Once leaf tissue was removed from the plant, it was immediately sliced into 2-mm width pieces with a razor blade, 0.2 g of FW was placed into test tubes with 10 mL of incubation medium containing 100 mM potassium phosphate buffer, 1 mM EDTA, and 1% (v/v) propanol at a pH of 7.5. Assay tubes were vacuum infiltrated twice at 450 mm Hg for 5 min to get a better accession of the assay solution into the cells. At once, leaf segments were incubated in the dark for one hour to inhibit nitrite reductase at 30 °C. Eventually, ${\text{NO}}_{2}^{-}$ released into the incubation medium was determined by adding in an orderly manner equivalent volumes of 1% sulfanilamide in HCl 1.5 N, and 0.1% Griess reagent (N-(1-naphthyl)-ethylenediamine hydrochloride) (Snell and Snell, 1949). The absorbance was determined at 540 nm once the colour was developed.

### 2.3 Data analysis

The data obtained either in the field or in the greenhouse experiments were analysed by the Mann–Whitney U test to compare the absence or the presence of BNI-trait in both ROELFS and MUNAL BNI-isogenic-lines. Additionally, for **Tables 3** and **4**, the data obtained in the greenhouse experiment were also analysed by one-way ANOVA using Duncan’s multiple range test for separation of means between N fertilization treatments. *p*-values< 0.05 were considered to be statistically significant differences.

## 3 Results

### 3.1 BNI-trait expression in ROELFS and MUNAL did not negatively impact grain yield or total dry matter production

Similar grain yield was found in Control and BNI wheat plants from ROELFS and MUNAL BNI-isogenic-lines (**Table 1**). Regarding crop biomass, no statistically significant differences were found between Control and BNI-isogenic-lines from ROELFS and MUNAL. Accordingly, the number of spikes m^-2^ were also akin between all BNI-isogenic-lines, with no differences in 1000-grain weight. However, there were some differences in the grain protein content, since it was 9% higher in ROELFS-BNI compared to its Control line; on the contrary, the MUNAL-BNI was 11% lower than its Control line.

**Table 1:** Grain yield, dry matter biomass, number of spikes, 1000-grain weight and grain protein of ROELFS and MUNAL BNI-isogenic-lines fertilized with 250 kg N ha^-1^ as ammonium sulphate.

	Grain Yield (kg ha^-1^)		Biomass (kg dry matter ha^-1^)		Spikes m^-2^		1000-grain weight (g)		Grain protein (%)	
ROELFS Control	5959 ± 355	a	11155 ± 559	a	204 ± 25	a	44.9 ± 0.7	a	13.0 ± 0.2	b
ROELFS BNI	5011 ± 99	a	10780 ± 82	a	221 ± 11	a	42.0 ± 0.1	a	14.2 ± 0.1	a
MUNAL Control	5487 ± 451	A	10556 ± 715	A	217 ± 13	A	45.6 ± 1.5	A	14.2 ± 0.1	A
MUNAL BNI	5504 ± 341	A	10854 ± 765	A	212 ± 16	A	46.3 ± 0.3	A	12.8 ± 0.0	B

The Mann-Whitney U test was used for the comparison between the absence or the presence of BNI-trait and the significant differences at p<0.05 are marked with a lowercase letter for ROELFS BNI-isogenic-line and capital letter for MUNAL BNI-isogenic-line.

### 3.2 ROELFS and MUNAL behaviour under different N sources

#### 3.2.1 BNI-trait expression at early stages of wheat growth

**Figure 1:**
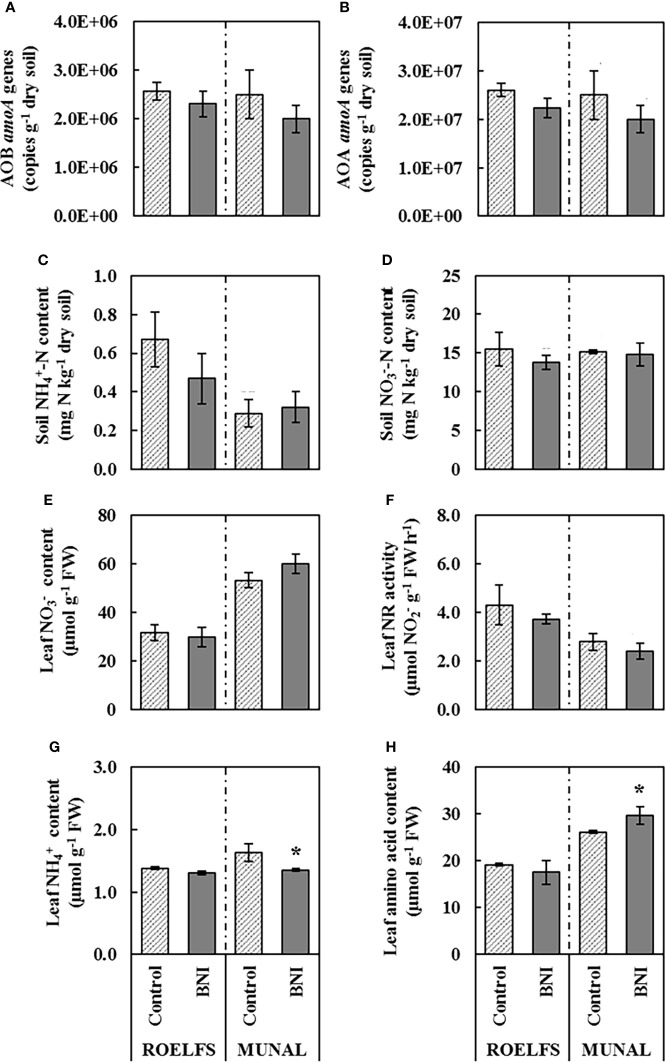
Performance of Control and BNI isogenic lines from ROELFS and MUNAL prior to fertilization (TO). Abundance of AOB **(A)** and AOA **(B)**, soil mineral nitrogen content such as ammonium **(C)** and nitrate **(D)**. and leaf determination of nitrate content **(E)**, nitrate reductase activity **(F)**, ammonium content **(G)**, and amino acid content **(H)**. The Mann-Whitney U test was used for the comparison between the absence or the presence of BNI-trait and the significant differences at p <0.05 are marked with an asterisk (*).

As it was expected, ROELFS-Control and ROELFS-BNI isogenic-lines did not present differences before the addition of N sources neither in the measured parameters of the soil (nitrifying microorganisms, ammonium, and nitrate) nor in those of the leaf (nitrate, ammonium, NR activity, and amino acids) (**Figure 1**). On the other hand, although there were no differences between MUNAL-Control and MUNAL-BNI isogenic-lines regarding the presence of nitrifying microorganisms (**Figures 1A, B**), soil mineral N (**Figures 1C, D**), leaf ${\text{NO}}_{3}^{-}$ content (**Figure 1E**), and leaf NR activity (**Figure 1F**), MUNAL-BNI plants showed less leaf ${\text{NH}}_{4}^{+}$ content (**Figure 1G**) and higher leaf amino acid content (**Figure 1H**) than MUNAL-Control plants when no N source was applied.

#### 3.2.2 BNI-wheat lines performance under ammonium fertilization

**Figure 2:**
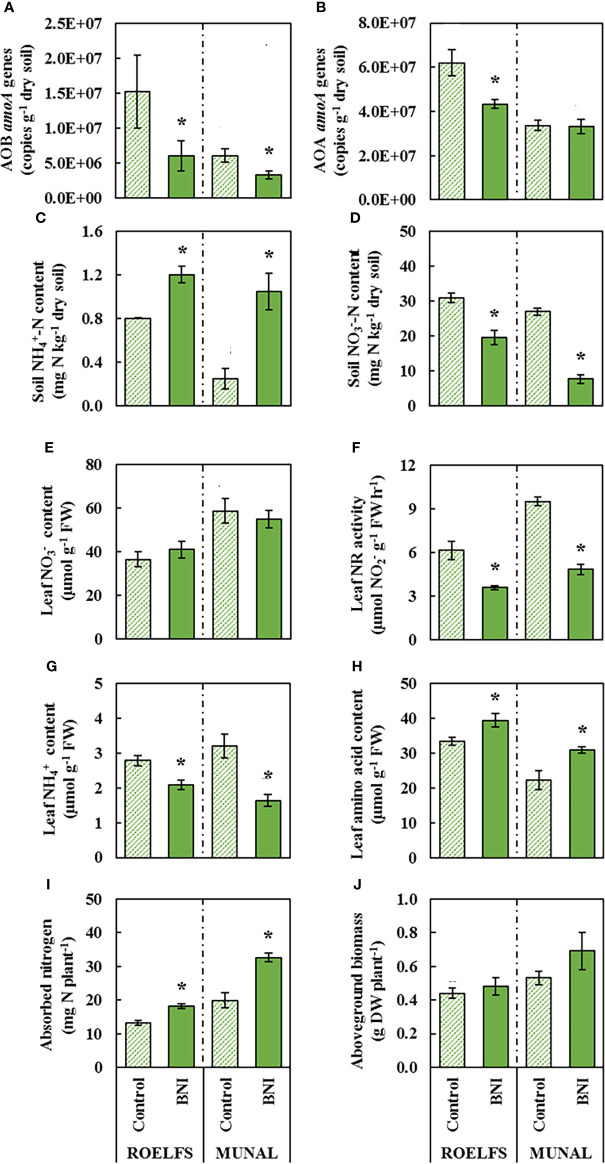
Performance of Control and BNI-isogenic-lines from ROELFS and MUNAL fertilized with **ammonium sulphate (AS)**. Soil and plant parameters were measured 30 days post-fertilization (T30): Abundance of AOB **(A)** and AOA **(B)**, soil mineral nitrogen content such as ammonium **(C)** and nitrate **(D)**, and leaf determination of nitrate content **(E)**, nitrate reductase activity **(F)**, ammonium content **(G)**, amino acid content **(H)**, absorbed nitrogen **(I)**, and aboveground biomass **(J)**. The Mann-Whitney U test was used for the comparison between the absence or the presence of BNI-trait and the significant differences at p <0.05 are marked with an asterisk (*).

**Figure 3:**
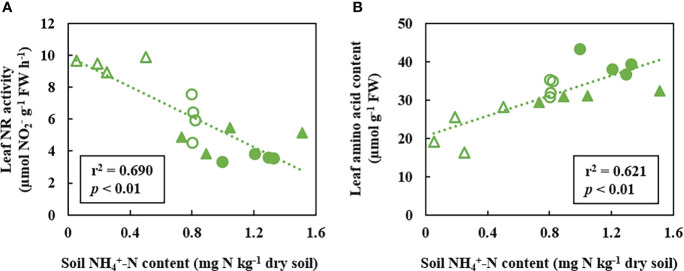
Correlation analysis between leaf NR activity **(A)** and leaf amino acid content **(B)** versus soil ${\text{NH}}_{4}^{+}-\text{N}$ content 30 days after fertilization with ammonium sulphate (AS). Empty circles correspond to ROELFS BNI isogenic-lines lacking BNI-trait (Control), full circles correspond to ROELFS BNI-isogenic-lines with BNI-trait (BNI), empty triangles correspond to MUNAL BNI-isogenic-lines lacking BNI-trait (Control), full triangles correspond to MUNAL BNI-isogenic-lines with BNI-trait (BNI).

In the first place, the application of N fertilization did not affect the abundance of total bacteria and archaea regardless of the N source in neither ROELFS nor MUNAL BNI-isogenic-lines (**Supplementary Figure 1**). However, after one month of ${\text{NH}}_{4}^{+}$ application, AOB growth was enhanced in soils where ROELFS-Control and MUNAL-Control plants grew (**Figure 2A**). Nevertheless, roots of ROELFS-BNI and MUNAL-BNI plants maintained the bacterial *amoA* abundance reduced in soil by 60% and 45%, respectively compared to their Control-isogenic-lines (**Figure 2A**). Moreover, while BNI-trait did not affect the AOA abundance in soil from MUNAL wheat plants, ROELFS-BNI plants were able to achieve a 30% reduction in AOA abundance respect to ROELFS-Control plants (**Figure 2B**). After 30 days of fertilization, the soil where ROELFS-BNI plants grew maintained 50% more ${\text{NH}}_{4}^{+}$ content compared to soil from ROELFS-Control plants (**Figure 2C**). This effect was more evident for MUNAL-BNI plants since soil was able to keep 4 times more ${\text{NH}}_{4}^{+}$ content compared to the soil from MUNAL-Control plants. Comparing soil ${\text{NO}}_{3}^{-}$ content, ROELFS-BNI and MUNAL-BNI plants were able to reduce nitrate by 44% and 74% in comparison to ROELFS-Control and MUNAL-Control plants respectively (**Figure 2D**). In leaves, although there were no differences regarding ${\text{NO}}_{3}^{-}$ content due to the BNI-trait neither in ROELFS nor MUNAL (**Figure 2E**), the leaf NR activity diminished 42% and 45% in ROELFS-BNI and MUNAL-BNI plants respectively (**Figure 2F**). The activity of leaf nitrate reductase has been described as a physiological indication of *in vivo* performance of BNI in *Brachiaria* (Karwat et al., 2019). In Control and BNI plants from both ROELFS and MUNAL BNI-isogenic-lines fertilized with ${\text{NH}}_{4}^{+}$ , a negative correlation between soil ${\text{NH}}_{4}^{+}$ content and leaf NR activity was observed (r^2^ = 0.690; *p*< 0.01; **Figure 3A**). Regarding the leaf ${\text{NH}}_{4}^{+}$ content, both ROELFS-BNI and MUNAL-BNI plants showed a 25% and 49% decrease compared to ROELFS-Control and MUNAL-Control plants (**Figure 2G**). ROELFS-BNI and MUNAL-BNI plants presented an 18% and 39% increase in the leaf amino acid content in comparison to their respective Control plants (**Figure 2H**). Moreover, we could also notice a positive correlation between leaf amino acid content and soil ${\text{NH}}_{4}^{+}$ content when considering Control and BNI plants from both ROELFS and MUNAL BNI-isogenic-lines (r^2^ = 0.621; *p*< 0.01; **Figure 3B**). The BNI-trait in both isogenic-lines increased the absorbed N by 36% of ROELFS-BNI and 64% in MUNAL-BNI plants (**Figure 2I**). There were no differences between Control and BNI plants in aboveground biomass (**Figure 2J**).

The abundance of denitrifying microorganisms, (**Supplementary Figure 2**) revealed no differences after 30 days of ammonium application. However, it is noticeable that ROELFS-BNI and MUNAL-BNI plants were able to decrease the *amoA*/*nirK* ratio by 63% and 39% respectively, compared to their Control plants (**Table 2**). In a similar way, the *amoA*/*nirK+nirS+nosZI+nosZII* ratio was diminished by 65% and 40% respectively compared to ROELFS-Control and MUNAL-Control plants. Nevertheless, the presence of the BNI-trait in ROELFS and MUNAL wheat did not affect significantly the balance between denitrifiers and complete denitrifiers.

**Table 2:** Gene ratio of bacterial nitrification and denitrification abundancies 30 days after fertilization with ammonium sulphate (AS).

	ROELFS		MUNAL	
	Control	BNI	Control	BNI
*amoA/nirK*	0.38	0.14* (-63%)	0.18	0.11* (-39%)
*amoA/nirK+nirS+nosZI+nosZII*	0.21	0.08* (-65%)	0.08	0.05* (-40%)
*nirK+nirS/nosZI+nosZII*	2.27	2.64	1.93	2.06

The Mann-Whitney U test was used for the comparison between the absence or the presence of BNI-trait and the significant differences at p<0.05 are marked with an asterisk (*).

#### 3.2.3 BNI-wheat lines performance under the combined effect of ammonium fertilization and DMPP

**Figure 4:**
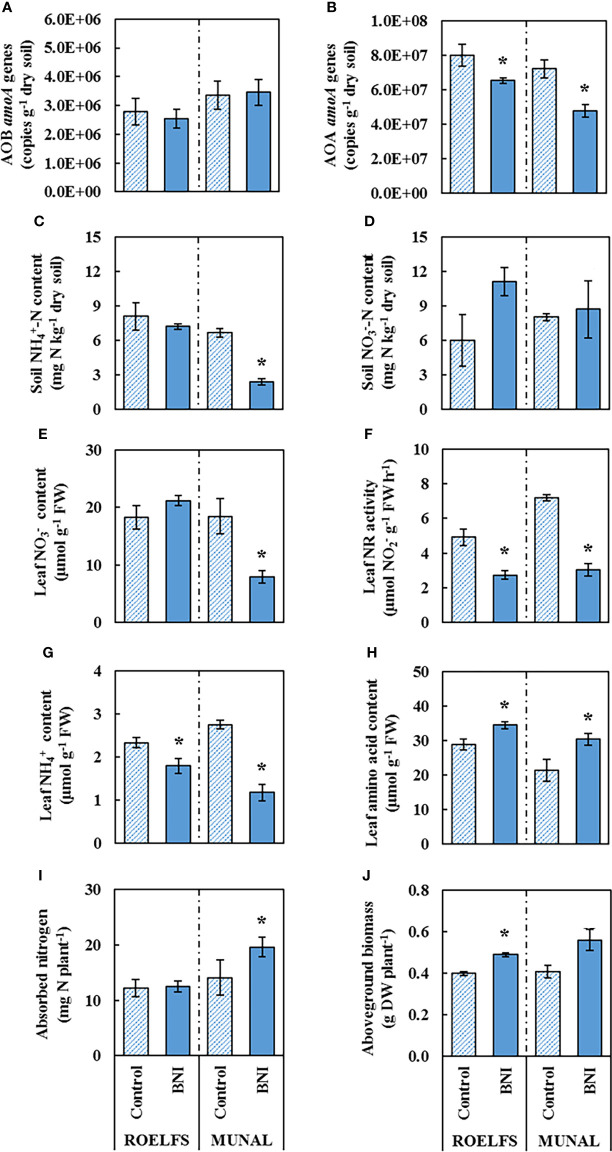
Performance of Control and BNI-isogenic-lines from ROELFS and MUNAL fertilized with **ammonium sulphate + DMPP (AS+D)**. Soil and plant parameters were measured 30 days post-fertilization (T30): Abundance of AOB **(A)** and AOA **(B)**, soil mineral nitrogen content such as ammonium **(C)** and nitrate **(D)**. and leaf determination of nitrate content **(E)**, nitrate reductase activity **(F)**. ammonium content **(G)**, amino acid content **(H)**, absorbed nitrogen **(I)**, and aboveground biomass **(J)**. The Mann-Whitney U test was used for the comparison between the absence or the presence of BNI-trait and the significant differences at p <0.05 are marked with an asterisk (*).

The application of an SNI, such as DMPP, enhanced the performance of BNI lines by keeping higher levels of soil-ammonium that is known to accelerate the synthesis and release of BNIs from root systems, including wheat (Subbarao et al., 2007b; Subbarao et al., 2007c); soil ammonium levels were 2-7 times higher in DMPP treatments combined with BNI genetic stocks (**Figure 2G**; **Tables 3**, **4**). The application of DMPP avoided the AOB increase in soils of both BNI-isogenic-lines of ROELFS and MUNAL (**Figure 4A**). DMPP was not able to inhibit the growth of AOA in the soil of Control plants (**Figure 4B**). Nevertheless, ROELFS-BNI and MUNAL-BNI plants were able to lower the AOA abundance, by 18% and 34%, respectively in presence of DMPP+AS. As commented, in general, DMPP maintained ammonium at maximum and the BNI-trait, in presence of DMPP, made ROELFS and MUNAL behave differently regarding soil ${\text{NH}}_{4}^{+}$ content (**Figure 4C**). MUNAL-BNI decreased it by 60% compared to MUNAL-Control; while both ROELFS-Control and ROELFS-BNI kept soil ${\text{NH}}_{4}^{+}$ content at maximum. There were no differences in soil ${\text{NO}}_{3}^{-}$ content between Control and BNI-isogenic-lines (**Figure 4D**).

**Table 3:** Statistical analysis of measured parameters in ROELFS Control and BNI-isogenic-lines.

	ROELFS					
	Control			BNI		
	AS	AS+D	KN	AS	AS+D	KN
AOB amoA genes (copies g^-1^ dry soil)	a	b	b	A*	B	B
AOA amoA genes (copies g^-1^ dry soil)	a	a	a	B*	A*	A
Soil ${\text{NH}}_{4}^{+}$ -N content (mg N kg^-1^ dry soil)	b	a	b	B*	A	C
Soil ${\text{NO}}_{3}^{-}$ N content (mg N kg^-1^ dry soil)	b	c	a	B*	C	A*
Leaf ${\text{NO}}_{3}^{-}$ content (µmol g^-1^ FW)	b	c	a	B	C	A
Leaf NR activity (µmol ${\text{NO}}_{2}^{-}$ g^-1^ FW h^-1^)	a	ab	b	B*	B*	A
Leaf ${\text{NH}}_{4}^{+}$ content (µmol g^-1^ FW)	a	b	c	A*	AB*	B
Leaf amino acid content (µmol g^-1^ FW)	a	b	c	A*	A*	B*
Absorbed nitrogen (mg N plant^-1^)	b	b	a	A*	C	B
Aboveground biomass (g DW plant^-1^)	b	b	a	A	A*	A

Significant differences between N treatments of Control isogenic lines are marked with a lowercase letter. Significant differences between N treatments of BNI isogenic lines are marked with a capital letter. The Mann-Whitney U test was used for the comparison between the absence or the presence of BNI-trait within the same fertilization treatment and the significant differences at p<0.05 are marked with an asterisk (*).

**Table 4:** Statistical analysis of measured parameters in MUNAL Control and BNI-isogenic-lines.

	MUNAL					
	Control			BNI		
	AS	AS+D	KN	AS	AS+D	KN
AOB amoA genes (copies g^-1^ dry soil)	a	b	b	AB*	A	B
AOA amoA genes (copies g^-1^ dry soil)	c	a	b	B	A*	A
Soil ${\text{NH}}_{4}^{+}$ -N content (mg N kg^-1^ dry soil)	b	a	b	B*	A*	C
Soil ${\text{NO}}_{3}^{-}$ N content (mg N kg^-1^ dry soil)	b	c	a	B*	BC	A*
Leaf ${\text{NO}}_{3}^{-}$ content (µmol g^-1^ FW)	a	b	a	B	C*	A*
Leaf NR activity (µmol ${\text{NO}}_{2}^{-}$ g^-1^ FW h^-1^)	a	b	c	B*	C*	A
Leaf ${\text{NH}}_{4}^{+}$ content (µmol g^-1^ FW)	a	a	a	A*	B*	A*
Leaf amino acid content (µmol g^-1^ FW)	a	a	a	A*	A*	A
Absorbed nitrogen (mg N plant^-1^)	a	b	a	A*	B*	A*
Aboveground biomass (g DW plant^-1^)	b	b	a	A	A	A

Significant differences between N treatments of Control isogenic lines are marked with a lowercase letter. Significant differences between N treatments of BNI isogenic lines are marked with a capital letter. The Mann-Whitney U test was used for the comparison between the absence or the presence of BNI-trait within the same fertilization treatment and the significant differences at p<0.05 are marked with an asterisk (*).

Fertilization with ${\text{NH}}_{4}^{+}$ combined with DMPP maintained similar leaf ${\text{NO}}_{3}^{-}$ content in ROELFS-Control and ROELFS-BNI isogenic-lines (**Figure 4E**). Contrarily, for MUNAL-BNI plants leaf ${\text{NO}}_{3}^{-}$ content was significantly lower compared to MUNAL-Control plants. Leaf NR activity was diminished by 45% and 58% respectively for ROELFS and MUNAL-BNI (**Figure 4F**). Similarly to ${\text{NH}}_{4}^{+}$ -only fertilization, the addition of DMPP also made ROELFS-BNI and MUNAL-BNI plants present lower leaf ${\text{NH}}_{4}^{+}$ content (**Figure 4G**) and higher leaf amino acid content (**Figure 4H**) compared to ROELFS-Control and MUNAL-Control plants. Lastly, ROELFS-BNI plants absorbed a similar N amount, but presented 23% higher aboveground biomass compared to ROELFS-Control plants (**Figures 4I, J**); in contrast, MUNAL-BNI plants absorbed 39% more N than MUNAL-Control plants without any differences in the aboveground biomass.

#### 3.2.4 BNI-wheat lines performance under nitrate fertilization

**Figure 5:**
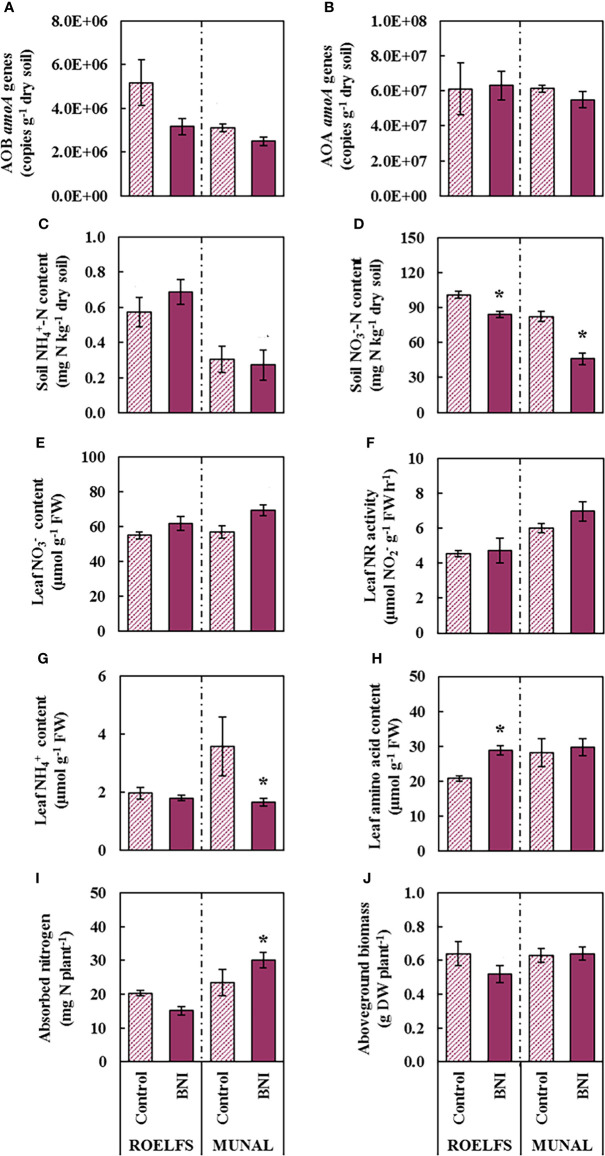
Performance of Control and BNI-isogenic-lines from ROELFS and MUNAL fertilized with **potassium nitrate (KN)**. Soil and plant parameters were measured 30 days post-fertilization (T30): Abundance of AOB **(A)** and AOA **(B)**, soil mineral nitrogen content such as ammonium **(C)** and nitrate **(D)**, and leaf determination of nitrate content **(E)**, nitrate reductase activity **(F)**, ammonium content **(G)**, amino acid content **(H)**, absorbed nitrogen **(I)**, and aboveground biomass **(J)**. The Mann-Whitney U test was used for the comparison between the absence or the presence of BNI-trait and the significant differences at p <0.05 are marked with an asterisk (*).

Fertilization with ${\text{NO}}_{3}^{-}$ had no effect on nitrifying microorganisms (**Figures 5A, B**), surely because the amount of ${\text{NH}}_{4}^{+}$ (**Figure 5C**) in the soil was minimal. Moreover, no differences in soil ${\text{NH}}_{4}^{+}$ content between any of the Control and BNI-isogenic-lines, ROELFS and MUNAL were detected (**Figure 5C**). Regarding soil ${\text{NO}}_{3}^{-}$ content, ROELFS-BNI and MUNAL-BNI plants were able to diminish it by 16% and 36%, respectively, and compared their respective control plants (**Figure 5D**). Regarding the genes analysed from the denitrification pathway (*nirK*, *nirS*, *nosZI*, and *nosZII*) the most noticeable changes occurred for MUNAL line. MUNAL BNI-lines showed 43% lower *nirK* abundance (**Supplementary Figure 2B**), and 48% lower *nosZI* abundance (**Supplementary Figure 2F**) compared to MUNAL-Control plants. Regarding the plants’ performance, there were no differences in leaf NO_3_- content and leaf NR activity (**Figures 5E, F**). On the other hand, MUNAL-Control plants presented higher leaf NH_4_^+^ content compared to MUNAL-BNI (**Figure 5G**), and no changes in ammonium occurred for ROELFS due to the BNI trait. Nonetheless, it was only ROELFS-BNI isogenic-line that presented 39% higher amino acids with respect to its control line (**Figure 5H**). Regarding the N absorbed, only MUNAL-BNI plants showed 28% more absorbed N than MUNAL-Control (**Figure 5I**). No differences in aboveground biomass between Control and BNI plants were observed for both lines (**Figure 5J**).

## 4 Discussion

### 4.1 BNI-trait does not entail metabolic costs on wheat productivity and/or grain yields

The biological nitrification inhibition (BNI) capacity of *L. racemosus* was recently demonstrated that could be transferred to elite wheat genetic stocks. Four elite wheat ROELFS, MUNAL, NAVOJOA, and QUAIU, carrying the T3BL.3Ns^b^S chromosome-region (Lr#-SA) of *L. racemosus* expressed the BNI trait with different abilities (Subbarao et al., 2021). ROELFS-BNI and MUNAL-BNI isogenic-lines were the two most successful lines in terms of expressing BNI capacity as both practically doubled the BNI activity released from roots compared to ROELFS-Control and MUNAL-Control plants. The introgression of BNI-trait into MUNAL wheat cultivar improved the grain yield in acidic soils (Subbarao et al., 2021); however, ROELFS-BNI showed lower grain yield compared to ROELFS-Control plants in three different trials in alkaline soils from Obregón (Subbarao et al., 2021). In the present work, the field experiment carried out in Mexico in 2019, ROELFS and MUNAL BNI-isogenic-lines presented similar agronomic characters. Consequently, we conclude that the acquisition of the BNI-trait by wheat does not entail a cost in metabolic terms of agronomic performance (**Table 1**). Furthermore, ROELFS and MUNAL were also grown in a microcosm experiment with a high fertilizer rate of different sources of N since wheat shows a relatively high tolerance to ammonium despite preferring nitrate as a source of N (González-Moro et al., 2021). Therefore, since no differences in yield of aboveground biomass between Control and BNI plants from ROELFS and MUNAL BNI-isogenic-lines were observed regardless of the N source (**Tables 3** and **4**), and they all presented a similar agronomic performance (**Table 1**) we can deduce that the cost of synthesizing BNI molecules does not affect wheat development and production.

The lack of difference in aboveground biomass and grain yield between Control and BNI plants might be attributed to the high fertilizer rates (200 kg N ha^-1^ for the microcosm’s experiment, and 250 kg N ha^-1^ for the field experiment) since as reported in Subbarao et al. (2021) the BNI-trait expresses its maximum potential at lower N inputs. Nevertheless, the presence of the BNI-trait could improve the N use efficiency (NUE) through nitrification inhibition, even with the addition of a high N fertilizer dose, as in the case of SNIs, where increases of 7% to 16% have been observed (IPCC, 2014; Kanter and Searchinger, 2018). Although ROELFS-BNI only showed higher N uptake under AS treatment (**Figure 2I**), MUNAL-BNI plants showed higher N uptake than MUNAL-Control regardless of the N source or the DMPP treatment (**Table 4**). The higher N absorption of MUNAL-BNI plants grown compared to ROELFS-BNI, observed in the pot experiment, could be possible because MUNAL-BNI plants showed higher BNI activity than ROELFS-BNI plants in hydroponic conditions (Subbarao et al., 2021). Even so, the plant N uptake from ROELFS-BNI and MUNAL-BNI was higher in treatments without DMPP and could represent an asset of BNIs over SNIs (**Tables 3**, **4**). Subbarao et al. (2021) hypothesized that the increase in N uptake is because the added Lr#-SA also carries genes that improve soil organic matter mineralization. BNI-harbouring wheat could displace the use of SNIs, which are not completely accepted by farmers as they have high costs without warranting better production (Subbarao et al., 2017). This affirmation is because the expression of the BNI trait would provide the plant with a more mixed N nutrition or a more balanced ${\text{NH}}_{4}^{+}{\text{/NO}}_{3}^{-}$ ratio in soil that would impulse crop yields (Subbarao and Searchinger, 2021). Nevertheless, although BNI-isogenic-lines from ROELFS and MUNAL achieved a higher N uptake, this did not translate into a higher grain protein content in the field. Since ROELFS-BNI plants presented a higher grain protein percentage than ROELFS-Control plants, meanwhile MUNAL-BNI showed less compared to its plants (**Table 1**), it seems that there are some differences between these wheat lines in grain protein remobilization and grain filling. Nevertheless, BNI-lines ROELFS and MUNAL grew well under the combined use of AS+DMPP, pointing out that the BNI trait could be linked to an “innate” capacity to assimilate ammonium as the primary source of N (**Figure 4J**). Accordingly, recent works related BNI release to ${\text{NH}}_{4}^{+}$ assimilation in the plant through plasma membrane H^+^-ATPase. This activity is entangled in the intracellular pH homeostasis through the release of protons to the rhizosphere (Afzal et al., 2020; Wang et al., 2021; Zhang et al., 2022). Thus, future studies are needed to understand the implications of BNI-trait on plant physiology and, from an agronomic point of view, on nitrogen uptake and yields under different wheat production environments and with different fertilizer rates, and how protein grain filling can be modified.

### 4.2 BNIs target the dominant nitrifying microorganisms in the soil

The ecological function of BNIs is to suppress soil nitrification by decreasing the ammonia-oxidizing microorganism populations (Subbarao et al., 2015). BNIs are known to have a stronger inhibitory impact on AOA rather than on AOB (Sarr et al., 2020; Kaur-Bhambra et al., 2022). However, the type of predominant nitrifying microorganism in soils is dependent on soil properties. In acidic soils, AOA are the predominant group carrying out the nitrification process, whereas AOB are relevant in neutral or alkaline soils (Nicol et al., 2008). Previously, the BNI-harboring MUNAL wheat was reported to reduce the AOA by 20% on acidic soils (Subbarao et al., 2021). Here, we show in alkaline soils that the BNI-trait clearly reduces the abundance of AOB (**Figure 2A**). The soil used in this pot experiment had a pH value of 8.0 (**Supplementary Table 1**), and it is classified as neutral-alkaline type according to the nature of most agricultural soils that surround the Mediterranean Sea area (Reuter et al., 2008; Fabian et al., 2014). Di and Cameron (2016) reported that in neutral-alkaline soils, AOA outnumber the abundance of AOB when ${\text{NH}}_{4}^{+}$ levels are low, but once ammonium-based fertilizers are applied, AOB outnumber AOA and become major players in carrying the nitrification process. As a result, soils tend to show an increase in bacterial *amoA* abundance (Castellano-Hinojosa et al., 2020; Bozal-Leorri et al., 2022). The expression of BNI-trait in ROELFS-BNI and MUNAL-BNI plants with ${\text{NH}}_{4}^{+}$ as the source of N seems to be effective against AOB in alkaline soils since they were able to reduce the *amoA* increment by 45-60% after ammonium fertilization (**Figure 2A**). Although nitrification is carried out mostly by AOB in neutral or alkaline soils (Nicol et al., 2008), AOA are still abundant, and, up to 10 times higher in the soil of this experiment (**Figures 1B**, **2B**, **4B** and **5B**). As BNIs exuded by ROELFS-BNI plants decreased the AOA abundance (**Figure 2B**), this result leads us to predict that BNIs from ROELFS would also target AOA in acidic soils, likewise as MUNAL (Subbarao et al., 2021), where they would be the main nitrifiers. Therefore, we hypothesize that BNIs could target the dominant nitrifying microorganism in any type of soil, which could widen the spectrum of soils and agrosystems where these kinds of wheat can be successfully cropped.

The application of SNIs, such as DMPP, inhibited ammonia-oxidizing bacteria (AOB) (**Figure 4A**; Bozal-Leorri et al., 2021; Corrochano-Monsalve et al., 2021), but its effect on AOA abundance remained unclear (Ruser and Schulz, 2015). In our case, BNI-trait presented a complementary or synergistic effect with SNIs. The application of DMPP did not affect the AOA abundance in soils where ROELFS-Control and MUNAL-Control plants (**Figure 4B**) were cultivated. Nevertheless, the combination of the SNI with the BNI-trait, made it possible to decrease the AOA abundance (**Figure 4B**). Furthermore, since both BNI-wheat lines maintain the abundance of total bacteria and archaea (**Supplementary Figure 1**), the effect of BNI would be specific for nitrifying microorganisms, and the impact on soil health would be minimal, as reported also for *Brachiaria* (Gopalakrishnan et al., 2009) where no negative impact on other microbes was observed. All in all, these results suggest that ROELFS-BNI and MUNAL-BNI plants could be very effective in reducing the nitrification process in agricultural soils, with pH spanning from acid to alkaline, irrespective of nitrifier population types, i.e. archaea or bacteria.

The inhibitory effect of ROELFS-BNI and MUNAL-BNI plants on soil ammonia-nitrifying microorganisms is reflected in the soil mineral N. The soil ${\text{NH}}_{4}^{+}$ content increases considerably after the application of ammonium-based fertilizers. However, when no synthetic nitrification inhibitors are added, ${\text{NH}}_{4}^{+}$ is oxidized in the first 30 days after fertilization (Torralbo et al., 2017). Accordingly, the efficiency of exudates with BNI activity from ROELFS-BNI and MUNAL-BNI plants in the slowdown in the nitrification process was doubly revealed, as they were able to maintain under AS treatment higher ${\text{NH}}_{4}^{+}$ content (**Figure 2C**) and lower ${\text{NO}}_{3}^{-}$ content in soils respect to ROELFS-Control and MUNAL-Control plants (**Figure 2D**). This behaviour is in line with the one described for MUNAL-BNI plants in acidic soils (Subbarao et al., 2021). Interestingly, under ${\text{NO}}_{3}^{-}$ nutrition BNI-isogenic-lines were effective in diminishing soil ${\text{NO}}_{3}^{-}$ content, especially MUNAL-BNI (**Figure 5D**). This may suggest that the expression of the BNI-trait in the plant could lead to a higher capacity to absorb and assimilate different N forms, thus, preventing its loss. The case of MUNAL-BNI, where higher amounts of absorbed N were observed, even with nitrate as the source of N, supports this hypothesis.

Denitrification is a process that depends on several soil environmental variables (Zumft, 1997), such as the level of anaerobiosis, modulated by soil moisture above 60% WFPS (Davidson, 1991) or nitrate content. In the present work, the soil moisture was adjusted to a 45% WFPS, which is optimal for nitrification, but not for denitrification. In view, there were no differences between soils from ROELFS-BNI and ROELFS-Control plants in the abundance of any of the denitrification genes analysed (*nirK, nirS, nosZI, nosZII*) after ammonium fertilization (**Supplementary Figure 2**). We suggest examining the effects of BNIs on denitrifying populations in soils with anaerobic conditions. Therefore, the lack of response from denitrifiers is the reason why we cannot see any difference in the *nirK*+*nirS*/*nosZI*+*nosZII* ratio when comparing both BNI-lines with their respective control-lines under SA fertilization (**Table 2**). Furthermore, the reduction in the nitrification/denitrification ratios stems from the capacity of BNI-isogenic-lines in reducing the bacterial *amoA* abundance, ROELFS-BNI being more efficient in inhibiting AOB growth in comparison to MUNAL-BNI plants (**Figure 2A**). On the other side, *nirK* and *nosZI* abundancies from KN-fertilized soils where MUNAL-BNI plants grew showed a decrease compared to those of MUNAL-Control plants (**Supplementary Figures 2B, F**). Although the effect of BNIs from pastures or crop plants on denitrifying populations has not been investigated, Florio et al. (2021) did state that denitrifiers’ abundance is not linked to the plant’s ability to exude BNIs, but rather to less formation of ${\text{NO}}_{3}^{-}$ , since soil ${\text{NO}}_{3}^{-}$ content is the substrate for denitrification. Thus, we hypothesise that the reduction of denitrifiers abundance in soil from MUNAL-BNI plants from KN treatment was caused by a reduction of soil ${\text{NO}}_{3}^{-}$ (**Figure 5D**), coupled with higher N uptake of MUNAL-BNI plants (**Figure 5I**).

### 4.3 The BNI-trait expression promotes adaptation to a more ammonium-based nutrition

Regarding a higher presence of soil ${\text{NH}}_{4}^{+}$ content, our results suggest that BNI-trait expression made wheat plants switch their metabolism towards a more ammonium nutrition. BNIs exuded by plants delay soil ${\text{NH}}_{4}^{+}$ oxidation, and decreased the formation of soil ${\text{NO}}_{3}^{-}$ and its plant uptake in AS treatment (**Figure 2**). Wheat is considered a relatively ammonium-tolerant plant (González-Moro et al., 2021) and performs quite well under ammonium fertilization (Fuertes-Mendizábal et al., 2013). As pointed above, BNI-lines grew better under the presence of SNI, perhaps owing to the transference of chromosome region Lr#-SA was able to induce a higher capacity to assimilate ammonium (**Figure 4J**). To evaluate the plant metabolism of BNI-harbouring wheat, we firstly determined the NR activity, which is a substrate-induced enzyme (Srivastava, 1980). Karwat et al. (2019) suggested leaf NR activity could be used as BNI-trait indicator for plants grown in greenhouse and field studies. The depletion of NR activity after ${\text{NH}}_{4}^{+}$ fertilization, even in presence of SNI, both in ROELFS and MUNAL (**Figures 2F**; **4F**) indicated the expression of BNI-trait by reduced soil nitrate production and induced the plant to use another N source different from nitrate. The negative response of NR activity to soil ${\text{NH}}_{4}^{+}$ content (r^2^ = 0.690; *p*< 0.01; **Figure 3A**) is interpreted in the sense that more ${\text{NH}}_{4}^{+}$ is maintained in the soil due to the presence of BNIs, and less ${\text{NO}}_{3}^{-}$ is absorbed, decreasing the NR activity in leaves. Despite that BNI lines maintained more soil ${\text{NH}}_{4}^{+}$ content (**Figures 2C**, **4C**), favouring ammonium nutrition compared to control plants, ROELFS-BNI and MUNAL-BNI plants kept less leaf ${\text{NH}}_{4}^{+}$ content (**Figures 2G**, **4G**). This lower leaf ammonium content could be explained because wheat behaves as an “ammonium excluder”, with a root-based mechanism. Therefore, similarly to other grasses, wheat copes with an ammonium excess by assimilating it in the roots using the carbon skeletons imported from shoots and, then, translocating the resulting amino acid to leaves (González-Moro et al., 2021). Furthermore, the strong correlation between leaf amino acid contents with soil ${\text{NH}}_{4}^{+}$ (r^2^ = 0.621; *p*< 0.01; **Figure 3B**) is interpreted in terms that many cereal species accumulate the ammonium into free amino acids content when they take up more ${\text{NH}}_{4}^{+}$ -N (González-Moro et al., 2021). Thus, the higher amino acid content in ROELFS-BNI and MUNAL-BNI plants is indicative of more ammonium nutrition due to the BNI activity. Thus, we suggest that the leaf amino acid content could be used as another indirect marker of plant BNI capacity. The more nitrification inhibition occurs, the higher is the ${\text{NH}}_{4}^{+}$ content in soils, which would result in a higher plant amino acid content. A similar effect on soil N mineral would be expected in the presence of the SNI, considering AOB and AOA populations depleted (**Figures 4A, B**). However, it must be noted that soil ${\text{NH}}_{4}^{+}$ contents were much more higher when DMPP was applied (**Tables 3**, **4**), meanwhile the soil ${\text{NO}}_{3}^{-}$ contents were lower (**Tables 3**, **4**), indicating the effectiveness of DMPP. As pointed above, BNI-lines grew also better or absorbed more N (**Figures 4I, J**) under the presence of SNI. Again, under these conditions, metabolic markers as NR activity and amino acid content indicated the higher capacity to assimilate ammonium-nitrogen by BNI lines (**Figures 4F, H**). Therefore, BNI-lines enhanced the ammonium assimilation when combined with DMPP, perhaps due to the transfer of the Lr#-SA chromosomal region, which was related to a greater capacity to assimilate ammonium as N source. In addition, the higher amino acid content in leaves of ROELFS-BNI plants fertilized with ${\text{NO}}_{3}^{-}$ (**Figure 5H**) could mean that even in presence of a N source that is different from ${\text{NH}}_{4}^{+}$ , i.e. ${\text{NO}}_{3}^{-}$ , the presence of the BNI trait primes an efficient use of N. As seen above, only ROELFS-BNI improved grain protein when fertilized with ${\text{NH}}_{4}^{+}$ (**Table 1**). Therefore, the recycling of N from aerial parts to grain or even the remobilization to root parts could differ between the lines studied. Nevertheless, these points would need further studies in the future.

## 5 Conclusion

The synthesis and release of BNI molecules had no metabolic cost for the studied wheat lines; Control- and BNI-isogenic-lines derived from ROELFS and MUNAL showed similar yield potential that include agronomic performance and plant development. The presence of the BNI-trait improved N absorption under ${\text{NH}}_{4}^{+}$ nutrition, and under ${\text{NO}}_{3}^{-}$ based fertilization in MUNAL-BNI plants. MUNAL-BNI plants were able to suppress AOB populations significantly, whereas ROELFS-BNI plants inhibited both AOB and AOA. Furthermore, when DMPP is added, MUNAL-BNI plants could suppress AOA populations also. Suppression of nitrifying populations resulted in higher soil- ${\text{NH}}_{4}^{+}$ levels and lowered soil- ${\text{NO}}_{3}^{-}$ levels. In this way, ${\text{NH}}_{4}^{+}$ -treated ROELFS-BNI and MUNAL-BNI plants showed a reduced leaf NR activity, due to a lower ${\text{NO}}_{3}^{-}$ uptake and transport to the leaf derived, consequently due to a decrease in soil ${\text{NO}}_{3}^{-}$ production. In sum, BNI-isogenic-lines appeared to shift the nitrogen metabolism to a more ammonium-based nutrition, with higher amino acid levels in leaf tissue compared to ROELFS-Control and MUNAL-Control plants. In addition, under ${\text{NO}}_{3}^{-}$ fertilization, ROELFS and MUNAL BNI-isogenic-lines also reduced soil ${\text{NO}}_{3}^{-}$ content. Thus, BNI-enabled elite wheat such as ROELFS-BNI and MUNAL-BNI, are part of BNI-technology, a newly emerging research area that can provide more efficient nitrogen technology for wheat production systems that are environmentally friendly and productive.

## Data availability statement

The raw data supporting the conclusions of this article will be made available by the authors, without undue reservation.

## Author contributions

AB-L, PA-T, CG-M and MBG-M conceived and designed the experiment. AB-L and LU performed the experiments. AB-L, GS, MK, LU, VK, HK, H-JB, PA-T, IO-M, CG-M and MBG-M analysed the data. AB-L wrote the first draft of the manuscript. All authors contributed to manuscript revision, read, and approved the submitted version.

## Funding

This project was funded by the Spanish Government (RTI2018-094623-B-C21 MCIU/AEI/FEDER, UE) and by the Basque Government (IT932-16; IT1560-22; 00048-ID2021-45). AB-L and LU held grants from the Basque Government (PRE-2020-2-0142 and PRE-2020-1-0127).

## Acknowledgments

The authors thank for the technical and human support of greenhouse provided by SGIker (UPV/EHU/ERDF, EU).

## Conflict of interest

The authors declare that the research was conducted in the absence of any commercial or financial relationships that could be construed as a potential conflict of interest.

## Publisher’s note

All claims expressed in this article are solely those of the authors and do not necessarily represent those of their affiliated organizations, or those of the publisher, the editors and the reviewers. Any product that may be evaluated in this article, or claim that may be made by its manufacturer, is not guaranteed or endorsed by the publisher.
