# Supplementary material for: Biological nitrification inhibitor-trait enhances nitrogen uptake by suppressing nitrifier activity and improves ammonium assimilation in two elite wheat varieties
Source: Front Plant Sci. 2022 Nov 11;13:1034219. doi: 10.3389/fpls.2022.1034219 (PMC9695736; doi:10.3389/fpls.2022.1034219)
Supplement: Supplementary file 1 [file Presentation_1.pptx]

## Slide 1
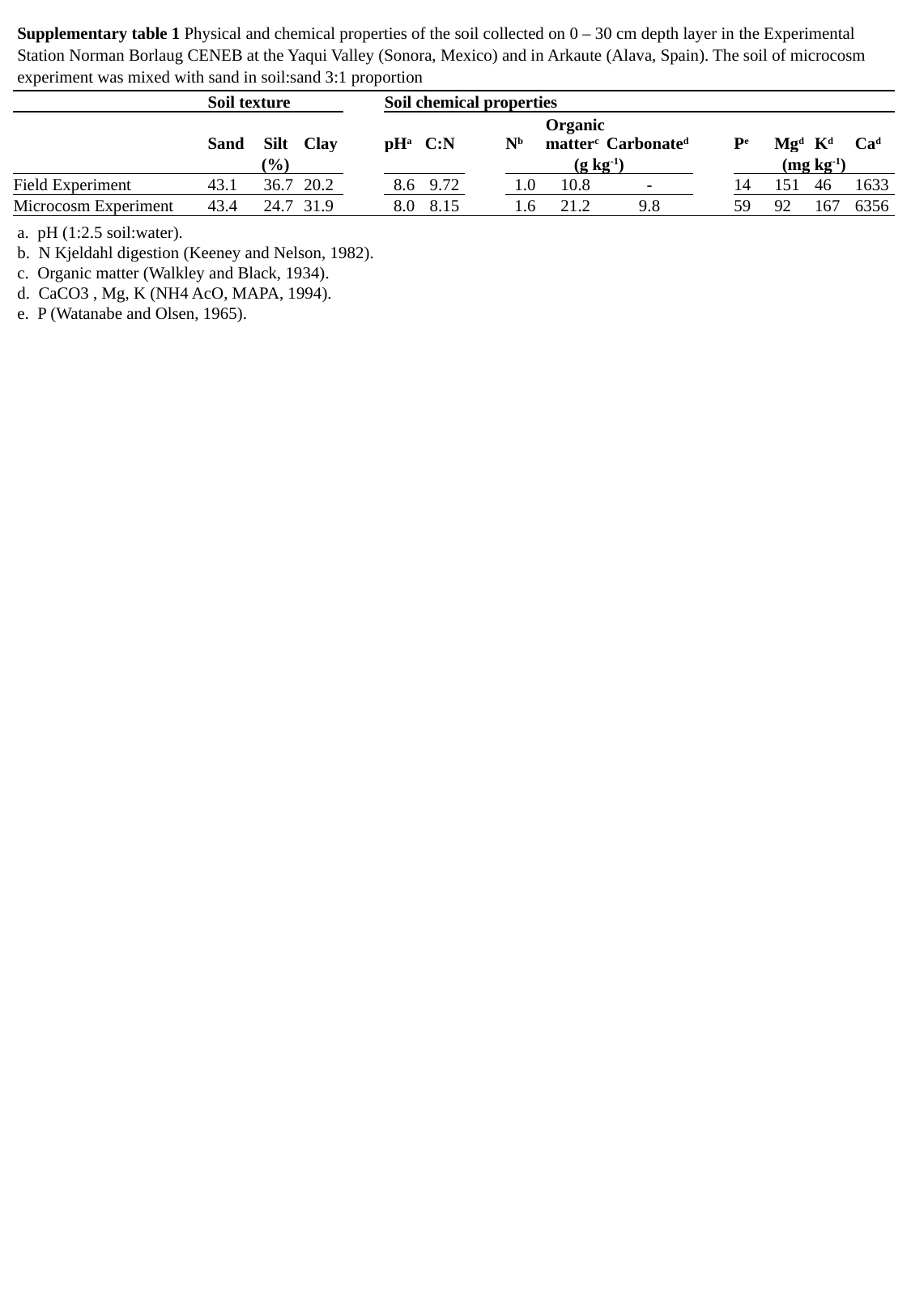

Supplementary table 1 Physical and chemical properties of the soil collected on 0 – 30 cm depth layer in the Experimental Station Norman Borlaug CENEB at the Yaqui Valley (Sonora, Mexico) and in Arkaute (Alava, Spain). The soil of microcosm experiment was mixed with sand in soil:sand 3:1 proportion
| | | Soil texture | | | | Soil chemical properties | | | | | | | | | | |
| --- | --- | --- | --- | --- | --- | --- | --- | --- | --- | --- | --- | --- | --- | --- | --- | --- |
| | | Sand | Silt | Clay | | pHa | C:N | | Nb | Organic matterc | Carbonated | | Pe | Mgd | Kd | Cad |
| | | (%) | | | | | | | (g kg-1) | | | | (mg kg-1) | | | |
| Field Experiment | | 43.1 | 36.7 | 20.2 | | 8.6 | 9.72 | | 1.0 | 10.8 | - | | 14 | 151 | 46 | 1633 |
| Microcosm Experiment | | 43.4 | 24.7 | 31.9 | | 8.0 | 8.15 | | 1.6 | 21.2 | 9.8 | | 59 | 92 | 167 | 6356 |
a.  pH (1:2.5 soil:water).
b.  N Kjeldahl digestion (Keeney and Nelson, 1982).
c.  Organic matter (Walkley and Black, 1934).
d.  CaCO3 , Mg, K (NH4 AcO, MAPA, 1994).
e.  P (Watanabe and Olsen, 1965).

## Slide 2
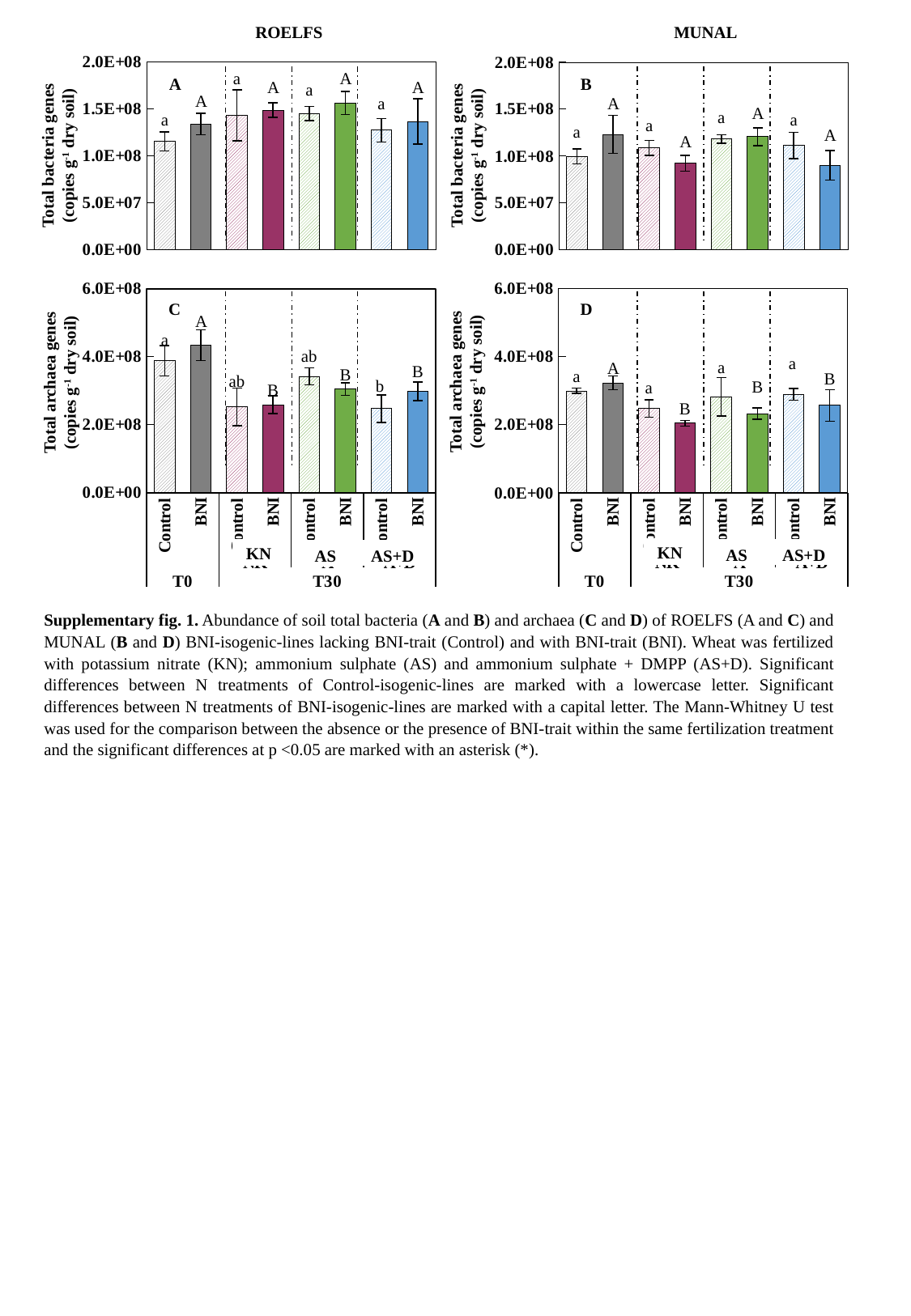

ROELFS
MUNAL
### Chart
| Category | |
|---|---|
| BNI- | 115030426.36829357 |
| BNI+ | 133759376.96246007 |
| BNI- | 142930752.36609945 |
| BNI+ | 148477228.7759863 |
| BNI- | 144982712.45270872 |
| BNI+ | 156265403.9696017 |
| BNI- | 127078758.41041134 |
| BNI+ | 136547549.5042766 |
### Chart
| Category | |
|---|---|
| BNI- | 99571709.59222972 |
| BNI+ | 123128300.9557538 |
| BNI- | 108661268.49916875 |
| BNI+ | 92066845.95574294 |
| BNI- | 118109184.42223512 |
| BNI+ | 120658251.66735817 |
| BNI- | 111238061.11137374 |
| BNI+ | 90165362.35117687 |A
B
Total bacteria genes
(copies g-1 dry soil)
Total bacteria genes
(copies g-1 dry soil)
### Chart
| Category | |
|---|---|
| Control | 388126822.15221214 |
| BNI | 433759376.96246004 |
| Control | 252293789.5410617 |
| BNI | 258946327.27583417 |
| Control | 342276482.5000159 |
| BNI | 304334911.1310067 |
| Control | 247256957.85864967 |
| BNI | 297765680.9578694 |
### Chart
| Category | |
|---|---|
| Control | 299571709.5922297 |
| BNI | 323128300.95575374 |
| Control | 247709047.49300775 |
| BNI | 204438092.98565283 |
| Control | 282289008.67114997 |
| BNI | 232562485.1059933 |
| Control | 289768544.1817028 |
| BNI | 256857730.50695786 |C
D
Total archaea genes
(copies g-1 dry soil)
Total archaea genes
(copies g-1 dry soil)
KN
KN
AS
AS+D
AS
AS+D
Supplementary fig. 1. Abundance of soil total bacteria (A and B) and archaea (C and D) of ROELFS (A and C) and MUNAL (B and D) BNI-isogenic-lines lacking BNI-trait (Control) and with BNI-trait (BNI). Wheat was fertilized with potassium nitrate (KN); ammonium sulphate (AS) and ammonium sulphate + DMPP (AS+D). Significant differences between N treatments of Control-isogenic-lines are marked with a lowercase letter. Significant differences between N treatments of BNI-isogenic-lines are marked with a capital letter. The Mann-Whitney U test was used for the comparison between the absence or the presence of BNI-trait within the same fertilization treatment and the significant differences at p <0.05 are marked with an asterisk (*).

## Slide 3
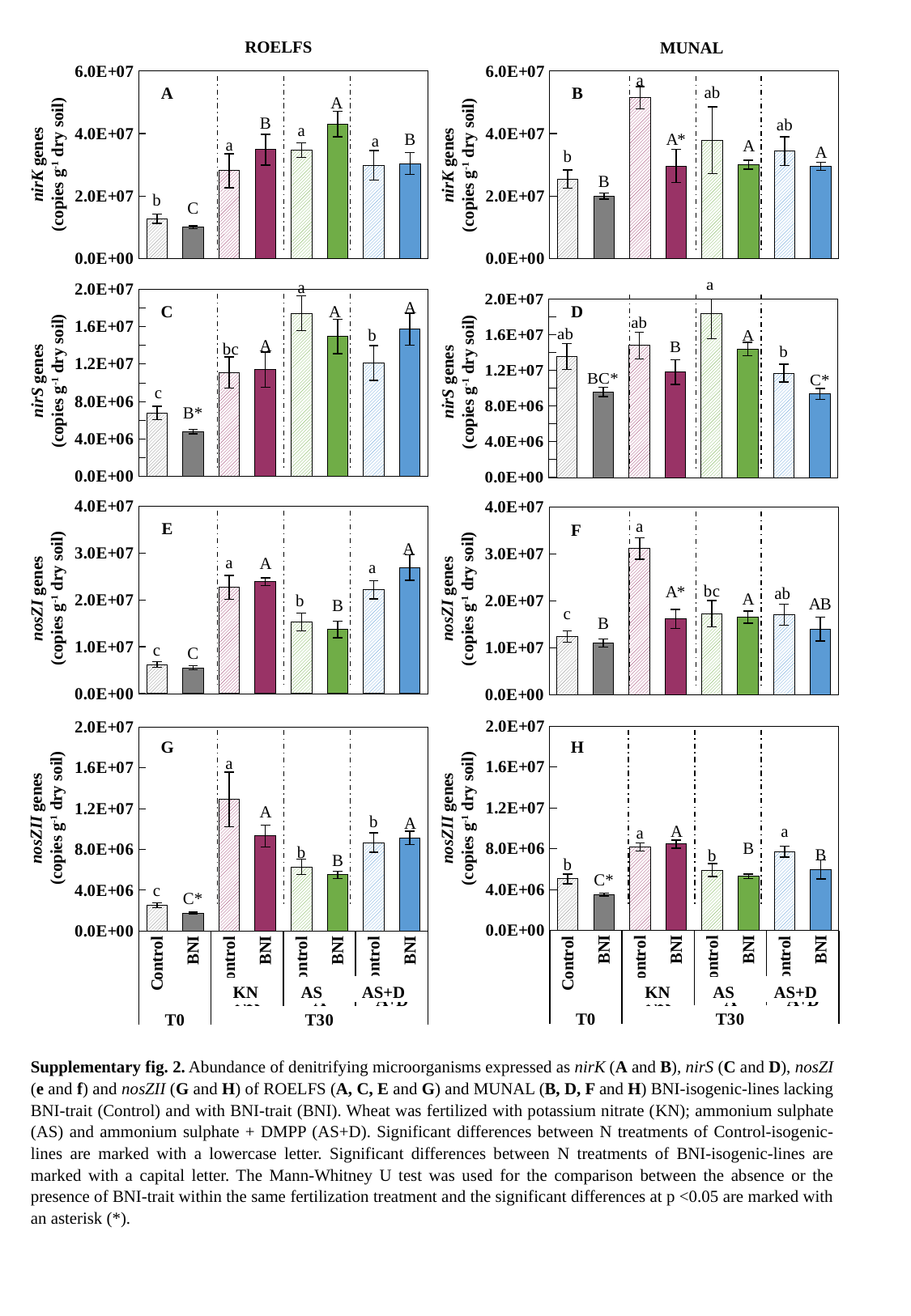

ROELFS
MUNAL
### Chart
| Category | |
|---|---|
| BNI- | 12715080.862292485 |
| BNI+ | 10006891.484661277 |
| BNI- | 28100139.899088476 |
| BNI+ | 34831795.94668667 |
| BNI- | 34663529.89932121 |
| BNI+ | 42997242.32928833 |
| BNI- | 29808091.828674372 |
| BNI+ | 30391778.336698644 |
### Chart
| Category | |
|---|---|
| BNI- | 25430161.72458497 |
| BNI+ | 20013782.969322555 |
| BNI- | 51501165.13381935 |
| BNI+ | 29605044.367567018 |
| BNI- | 37854874.26887287 |
| BNI+ | 30049502.977079686 |
| BNI- | 34358274.98455025 |
| BNI+ | 29501906.9476303 |A
B
nirK genes
(copies g-1 dry soil)
nirK genes
(copies g-1 dry soil)
### Chart
| Category | |
|---|---|
| BNI- | 6784023.050935905 |
| BNI+ | 4798327.944250595 |
| BNI- | 11090579.357365994 |
| BNI+ | 11408684.464369668 |
| BNI- | 17421226.003682446 |
| BNI+ | 14939556.444215633 |
| BNI- | 12112211.354605768 |
| BNI+ | 15716246.197741961 |
### Chart
| Category | |
|---|---|
| BNI- | 13568046.10187181 |
| BNI+ | 9596655.88850119 |
| BNI- | 14792307.165250104 |
| BNI+ | 11806078.324799588 |
| BNI- | 18324319.63010759 |
| BNI+ | 14408807.177211383 |
| BNI- | 11688404.560453339 |
| BNI+ | 9376732.87206604 |C
D
nirS genes
(copies g-1 dry soil)
nirS genes
(copies g-1 dry soil)
### Chart
| Category | |
|---|---|
| BNI- | 6187239.102245592 |
| BNI+ | 5498970.917793223 |
| BNI- | 22657713.72360316 |
| BNI+ | 23849490.425737277 |
| BNI- | 15268168.897203596 |
| BNI+ | 13662843.241982587 |
| BNI- | 22134748.096756484 |
| BNI+ | 26881988.274724048 |
### Chart
| Category | |
|---|---|
| BNI- | 12374478.204491183 |
| BNI+ | 10997941.835586445 |
| BNI- | 31167901.25777953 |
| BNI+ | 16122135.548983647 |
| BNI- | 17234948.13618032 |
| BNI+ | 16512754.55703568 |
| BNI- | 17026160.17946689 |
| BNI+ | 13945742.165968671 |E
F
nosZI genes
(copies g-1 dry soil)
nosZI genes
(copies g-1 dry soil)
### Chart
| Category | |
|---|---|
| Control | 5044102.132316411 |
| BNI | 3520880.099824286 |
| Control | 8171486.592729969 |
| BNI | 8464084.867019357 |
| Control | 5906807.943409559 |
| BNI | 5286391.743319267 |
| Control | 7726770.508938876 |
| BNI | 5967608.767145752 |
### Chart
| Category | |
|---|---|
| Control | 2522051.0661582053 |
| BNI | 1760440.049912143 |
| Control | 12902308.426726468 |
| BNI | 9315725.54006814 |
| Control | 6282714.193668077 |
| BNI | 5503251.593575003 |
| Control | 8673370.23281203 |
| BNI | 9143917.587399095 |G
H
nosZII genes
(copies g-1 dry soil)
nosZII genes
(copies g-1 dry soil)
AS
AS
AS+D
AS+D
KN
KN
Supplementary fig. 2. Abundance of denitrifying microorganisms expressed as nirK (A and B), nirS (C and D), nosZI (e and f) and nosZII (G and H) of ROELFS (A, C, E and G) and MUNAL (B, D, F and H) BNI-isogenic-lines lacking BNI-trait (Control) and with BNI-trait (BNI). Wheat was fertilized with potassium nitrate (KN); ammonium sulphate (AS) and ammonium sulphate + DMPP (AS+D). Significant differences between N treatments of Control-isogenic-lines are marked with a lowercase letter. Significant differences between N treatments of BNI-isogenic-lines are marked with a capital letter. The Mann-Whitney U test was used for the comparison between the absence or the presence of BNI-trait within the same fertilization treatment and the significant differences at p <0.05 are marked with an asterisk (*).
